# Supplementary material for: Deprivation Index and Lifestyle: Baseline Cross-Sectional Analysis of the PREDIMED-Plus Catalonia Study
Source: Nutrients. 2021 Sep 27;13(10):3408. doi: 10.3390/nu13103408 (PMC8540452; doi:10.3390/nu13103408)
Supplement: Supplementary file 1 [file nutrients-13-03408-s001.zip › nutrients-1355952-supplementary.pdf]

Table S1. Food groups

**Food group**	**Items**
Full-fat dairy	*Whole milk, yoghurt (whole), petite-suisse, cottage cheese and curd, semicured/cured cheeses fresh cheese, milkshakes, custard, flan and pudding, ice cream.*
Low-fat dairy	*Semi-skimmed milk, non-fat milk, skimmed yoghurt*
White meat	*Chicken or turkey with skin, chicken or turkey without skin, rabbit*
Red meat and meat products	*Beef or veal meat, pork meat, lamb meat, liver, entrails, cured ham, boiled ham, blood sausage, pâté, hamburgers, bacon*
Eggs	*Egg*
Fish and seafood	*White fish, blue fish, salad fish, oysters, clams and mussels, squid, octopus, prawn, king prawn, crayfish, fatty fish canned in oil; fatty fish canned in salted water.*
Refined cereals	*White bread, breakfast cereals, white rice, pasta (spaghetti, macaroni, noodles, others), pizzas*
Whole-grain cereals	*Whole-grain bread, whole-grain cereals, muesli, oat grains, all bran, brown rice, whole-wheat pasta, whole-grain and fiber biscuits.*
Legumes	*Lentils, chickpeas, beans and peas*
Fruits	*Oranges, tangerines, grapefruits, bananas, apples and pears, strawberries, cherries and plums, watermelon, melon, kiwi, grapes, raisins*
Vegetables	*Swiss chards and spinach, cabbage, cauliflower, broccoli, lettuce, endive, tomato, carrot and pumpkin green bean, zucchini, aubergines and cucumbers, peppers, asparagus, "gazpacho", onions, garlic, mushrooms and champignons, other vegetables*
Nuts	*Almonds, pistachios, hazelnuts and other nuts*
Olive oil and olives	*Olives, refined olive oil, extra virgin olive oil*
Other fat or oils, full-fat dairy derivatives and processed meal	*Olive pomace oil, sunflower oil, corn oil, soy oil, other oils, butter and lard, margarine, condensed milk, cream, croquettes and packet soup or creamy soup, mustard, mayonnaise, tomato sauce, others snacks than French fries*
Potatoes	*Boiled or roast potatoes and French fries*
Biscuits and pastries	*Simple cookies, chocolate cookies, home-made bakery, industrial bakery, doughnuts, muffins.*
Sugar, sweets, chocolate and cocoa	*Fruits in syrup, sugar, jam and honey, chocolate and chocolates, cocoa powder*
Sugary beverages and juices	*Sugar-sweetened soft drinks, natural orange juice, natural other fruits juices and bottled fruit juices*
Sugar-free beverages	*Sugar-free beverages*
Coffee and tea	*Coffee, decaffeinated coffee, tea*
Cava and beers	*Cava, beers*
Spirits beverages	*Liqueurs, anis and anisette, whiskey, gin, vodka*
Wines	*Musts, rosé wine, muscatel wine, young red wine, old red wine, white wine*

Table S2. Relationship (multivariable adjusted β-coefficients and 95%CI) between CSI and food groups consumption

	**β**	**[95% CI]**	***p-value***
Full-fat dairy (g/d)	-6.67	[-16.51;3.18]	0.184
Low-fat dairy (g/d)	-12.39	[-30.69;5.92]	0.185
White meat (g/d)	0.17	[-3.22;3.57]	0.920
Red meat and meat products (g/d)	-3.17	[-7.32;0.99]	0.135
Eggs (g/d)	1.63	[-0.61;3.87]	0.230
Fish and seafood (g/d)	0.54	[-4.11;5.18]	0.821
Whole grain cereals (g/d)	1.97	[-3.72;7.67]	0.497
Refined cereals (g/d)	11.98	[5.24;18.72]	0.001
Legumes (g/d)	0.66	[-0.4;1.72]	0.224
Fruits (g/d)	-17.52	[-33.89; -1.14]	0.036
Vegetables (g/d)	0.88	[-12.23;13.99]	0.895
Nuts (g/d)	-0.18	[-1.62;1.27]	0.811
Olive oil and olives (g/d)	-0.21	[-2.21; 1.78]	0.834
Other fat or oils, full-fat dairy derivatives and processed meal (g/d)	-0.06	[-1.11;1]	0.918
Potatoes (g/d)	6.68	[2.76;10.61]	0.001
Biscuits and pastries (g/d)	0.66	[-0.4;1.72]	0.154
Sugar, sweets, chocolate and cocoa (g/d)	0.21	[-1.56;1.99]	0.814
Sugary beverages and juices (g/d)	-5.2	[-16.63;6.23]	0.373
Sugar-free beverages (g/d)	-10.46	[-21.87;0.95]	0.072
Coffee and tea (g/d)	-8.03	[-14.34; -1.72]	0.013
Cava and beers (g/d)	1.73	[-15.46;18.92]	0.844
Spirits beverages (g/d)	0.15	[-0.93;1.22]	0.789
Wines (g/d)	-5.00	[-12.75;2.75]	0.206

Multiple linear regressions. Beta-coefficient and [95% CI] are shown. CSI (low/high deprivation index) as exposure and food consumption (food groups, g/d) as outcome, adjusted by age (years), sex (men/women), smoking (smoker, former or never smoked), waist circumference (cm), physical activity (low/high), sedentary lifestyle (no/yes), hypertension (no/yes), dyslipidemia (no/yes) and type 2 diabetes mellitus.

Table S3. Dietary patterns and food groups

	**Healthy pattern n=704**	**Unhealthy pattern n=808**	***p-value#***
Full-fat dairy (g/d)	51.1 [29.9;87.0]	75.2 [42.6;136]	<0.001
Low-fat dairy (g/d)	241 [139;336]	196 [49.2;263]	<0.001
White meat (g/d)	74.8 [62.9;86.6]	64.8 [32.1;76.1]	<0.001
Red meat and meat products (g/d)	84.8 [61.6;113]	92.5 [69.9;122]	<0.001
Eggs (g/d)	25.2 [22.3;26.6]	25.0 [21.7;26.8]	0.823
Fish and seafood (g/d)	131 [100;162]	88.4 [62.6;117]	<0.001
Whole grain cereals (g/d)	59.3 [2.43;83.2]	0.72 [0.00;21.5]	<0.001
Refined cereals (g/d)	69.2 [36.1;107]	133 [96.4;191]	<0.001
Legumes (g/d)	20.2 [14.4;26.5]	15.9 [12.4;21.5]	<0.001
Fruits (g/d)	357 [277;486]	241 [160;322]	<0.001
Vegetables (g/d)	369 [297;468]	257 [202;321]	<0.001
Nuts (g/d)	14.2 [6.03;28.9]	6.50 [2.93;12.9]	<0.001
Olive oil and olives (g/d)	57.7 [47.6;67.7]	54.5 [43.4;65.4]	<0.001
Other fat or oils, full-fat dairy derivatives and processed meal (g/d)	5.03 [2.32;9.43]	8.03 [3.96;14.1]	<0.001
Potatoes (g/d)	89.5 [41.0;100]	92.8 [49.7;106]	<0.001
Biscuits and pastries (g/d)	12.5 [4.89;21.2]	17.4 [7.74;32.4]	<0.001
Sugar, sweets, chocolate and cocoa (g/d)	12.2 [5.08;21.8]	17.7 [7.59;30.9]	<0.001
Sugary beverages and juices (g/d)	34.7 [9.07;106]	40.0 [10.5;119]	0.229
Sugar-free beverages (g/d)	1.34 [0.00;13.4]	1.60 [0.00;12.7]	0.520
Coffee and tea (g/d)	76.9 [48.4;127]	101 [49.6;128]	0.058
Cava and beers (g/d)	38.1 [3.89;78.9]	64.4 [21.0;155]	<0.001
Spirits beverages (g/d)	0.77 [0.00;2.10]	1.50 [0.00;3.86]	<0.001
Wines (g/d)	20.7 [3.44;51.2]	35.9 [9.09;96.5]	<0.001

Data are presented as median [IR].
p-value#: Mann–Whitney U test
